# Effects of exercise therapy for pregnancy-related low back pain and pelvic pain: A protocol for systematic review and meta-analysis

**DOI:** 10.1097/MD.0000000000017318

**Published:** 2020-01-17

**Authors:** Xiang Hu, Ming Ma, Xianghu Zhao, Wudong Sun, Yanli Liu, Zengbin Zheng, Liang Xu

**Affiliations:** a Department of Rehabilitation, Zhongda Hospital Affiliated to Southeast University, Nanjing, Jiangsu; b Center of Rehabilitation, Wuhan Polytechnic University, Wuhan, Hubei, China.

**Keywords:** exercise, meta-analysis, patients, pelvic pain, pregnancy-related low back pain, systematic review

## Abstract

**Background:**

Pregnancy-related low back pain (PLPB) and pelvic pain (PP) are common in pregnancy. In spite of its high prevalence rate, treatment of the disorder is a challenging topic. Women commonly utilize complementary exercise therapies such as yoga, motor control exercises, breathing exercises, core stability exercise, pelvic stability exercise, and so on to manage their symptoms. However, it is currently unknown whether exercise produces more beneficial effects than other treatment in patients with PLPB and PP. The aim of this study is to explore the therapeutic effect of exercise for pregnancy-related low back pain and PP.

**Methods:**

This review will only include randomized controlled trials. Published articles from July 1999 to July 2019 will be identified using electronic searches. Search strategy will be performed in 3 English databases, 1 Chinese database, and the World Health Organization International Clinical Trials Registry Platform. Two reviewers will screen, select studies, extract data, and assess quality independently. The methodological quality including the risk of bias of the included studies will be evaluated using a modified assessment form, which is based on Cochrane assessment tool and Physiotherapy Evidence Database scale. Review Manager Software (Revman5.3) will be used for heterogeneity assessment, generating funnel-plots, data synthesis, subgroup analysis, and sensitivity analysis. We will use GRADE system to evaluate the quality of our evidence.

**Results:**

We will provide some more practical and targeted results investigating the effect of exercise therapy (ET) for PLPB and PP in the current meta-analysis. Meanwhile, we will ascertain study progress of ET for PLPB and PP and find out defects or inadequacies of previous studies, so that future researchers could get beneficial guidance for more rigorous study.

**Conclusion:**

The stronger evidence about PLPB and PPs rehabilitative effect and safety will be provided for clinicians and policymakers.

**Systematic review registration:**

PROSPERO CRD 42017075099.

## 1 Introduction

### 1.1 Description of the condition

Pregnancy-related low back pain (PLBP) and pelvic pain (PP) are very common musculoskeletal pain during pregnancy. It can be caused affect daily activities such as walking, work, sleep, mood, and so on, consequently reducing the quality of life, and there is some evidence of socioeconomic detriment, mainly due to absence from work.^[1,2]^

LBP is usually defined as pain between the twelfth rib and the gluteal fold, whereas PP is defined as pain experienced between the posterior iliac crest and the gluteal fold, particularly in the vicinity of the sacroiliac joints. Previous studies have reported that 25% of newly delivered women and 44% of pregnant women experienced low back pain and PP, or both.^[3]^ Although most women recover within a month after delivery, a significant percentage (5%–8.5%) continue to complaints even up within 2 years after delivery.^[4]^ To improve patients’ functional status and quality of life, it is important to understand which structures are capable of producing pain and disability. But the exact cause(s) of pregnancy-related LBP and PP is not clear.^[5]^ Current studies suggest that pregnancy-related LBP and PP may be associated with mechanical factors, mainly due to weight gain and postural changes during pregnancy, lead to body center of gravity moved forward increased lumbar processes, and increased pressure on the lower back.^[6,7]^ Pelvic floor dysfunction is closely related to LBP. The negative active straight leg elevation test and the positive post-pain stimulation test can be interpreted as increased pelvic floor muscle activity to compensate for impaired pelvic stability. Meanwhile, hormonal changes and relaxin increases can lead to inefficient neuromuscular control, ligament relaxation and discomfort, not only in the sacroiliac joint, but also in general discomfort, whole back pain, pelvic instability, and spine dislocation during pregnancy.^[6–8]^ Considering each woman's personality and pregnancy situation, early detection and treatment will lead to the best possible results. Thus, in order to gain muscle strength, flexibility, and endurance, to restore injured tissues, and to contribute to ability to sustain normal life activities, exercise is one of the most frequently used modalities in the rehabilitation of subjects with pregnancy-related LBP and PP.

Considering the above reasons, the purpose of this systematic review was to investigate the effect of exercise therapy (ET) on pregnancy-related LBP and PP.

### 1.2 Description of intervention

European guidelines recommend that PLBP and PP, are managed by providing information and patients are advised to maintain a positive attitude, encouraged to continue their normal daily activities and work as much as possible and given individual exercises as appropriate.^[7]^ In recent years, Core stability training has become a popular fitness trend that has begun to be applied in rehabilitation programs and in sports medicine.^[9]^ Many studies have examined the effects of a stabilization exercise program involving training of the pelvic floor and abdominal muscles to increase compressive forces in the SI joints. Some studies have shown positive results on pain reduction.^[10–12]^ However, there is room for improvement due to:

(1) unsatisfactory results in more than 6% to 40% of women^[13–15]^;
(2) the long time (4–12 weeks) required to achieve pain reduction^[16]^; and
(3) lack of evidence of the effects of stabilization on the alignment and conformity of the SI joints.^[17]^

Mens et al^[18]^ found no differences in peripartum PP between an exercise group performing diagonal trunk muscle exercises and a control group. On the other hand, Stuge et al^[11]^ found that specific stabilizing exercise for 20 weeks postpartum was more effective than general care including massage, relaxation, joint mobilization and strengthening exercises. Moreover, a longer-term intervention study with a 2-year follow-up showed that a specific stabilization exercise was more effective than a control group.^[19]^ A systematic review of 5 selected studies on the effects of an exercise program for postpartum pain, involving an intervention period of between 4 and 20 weeks, concluded that the exercises were effective. Low back and pelvic exercises were introduced as a rehabilitation program to limit pain, maximize function, and prevent further injury.^[20]^ This is accomplished through a series of exercises that are relatively simple with respect to time and equipment, but are physiologically complex. Despite the popularity of stabilization training in the treatment of back and PP,^[21]^ However, it is currently unclear whether exercise produces more beneficial effects than other treatments for patients with PLBP and PP.

### 1.3 Objective of this study

The objective of our study was to review all observational studies or clinical studies of patients with PLBP and PP treated using various exercises therapy compared with other techniques to relieve the pain when used for this purpose.

## 2 Methods

This review protocol has been registered in the PROSPERO, which is the International Prospective Register of systematic reviews. Its registration number was CRD42017075099. Cochrane Handbook of Systematic Reviews of Interventions (Version 5.1.0, http://www.cochranehandbook.org) will guide this systematic review. The statement of preferred reporting items for systematic review and meta-analysis protocols (PRISMA-P)^[22]^ and PRISMA^[23]^ will be used as guidelines for reporting present review protocol and the formal paper that follows. This protocol for systematic review and meta-analysis comes from published data and does not involve patients, so no ethical approval is required.

### 2.1 Inclusion criteria for study selection

#### 2.1.1 Types of studies

Only randomized controlled trials (RCTs) will be included, whereas non-RCTs, quasi-RCTs, and any other types of studies will be excluded.

#### 2.1.2 Types of participants

In our study, participants will be diagnosed as PLBP and PP regardless of their age, or race.

#### 2.1.3 Types of interventions

We will include articles comparing treatment groups which received ET. The ET program can be described as enhancing the ability to ensure a stable neutral spine position.^[9]^ Exercises are usually nonmedication form of physical therapy such as aerobic exercise, stability exercise, strength exercise, and so on.^[20]^

#### 2.1.4 Types of outcome assessments

In our study, primary outcomes will include pain score. Secondary outcomes will include low back and pelvic function and disability, health-related quality of life, and adverse events.

#### 2.1.5 Search strategy

To avoid losing any available literature that might meet our needs, we will systematically search the following electronic databases: PUBMED, The Cochrane Library, EMBASE, China Biology Medicine disc. All English and Chinese literature, published from July 1, 1999 to July 1, 2019, will seek to be unrestricted by race, gender or region. Our search will also include the World Health Organization International Clinical Trial Registry Platform and its Registry Network for additional unpublished or ready to be published studies. In addition, the list of references to previous clinical studies and reviews will be served as the searching object. Search strategies will be established according to the Cochrane handbook. PUBMED's search strategy is shown in Table 1, and similar search strategies will be used for other electronic databases.

**Table 1:**
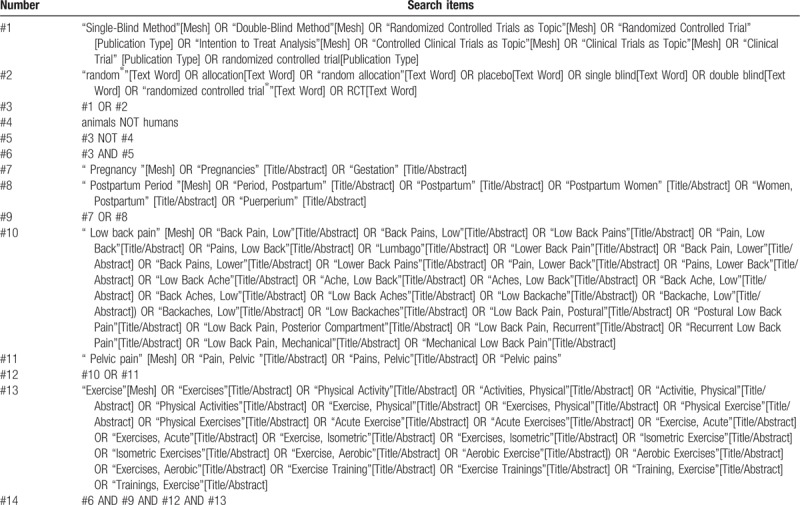
Search strategy for PUBMED.

### 2.2 Data collection and analysis

#### 2.2.1 Selection of studies

**Figure 1:**
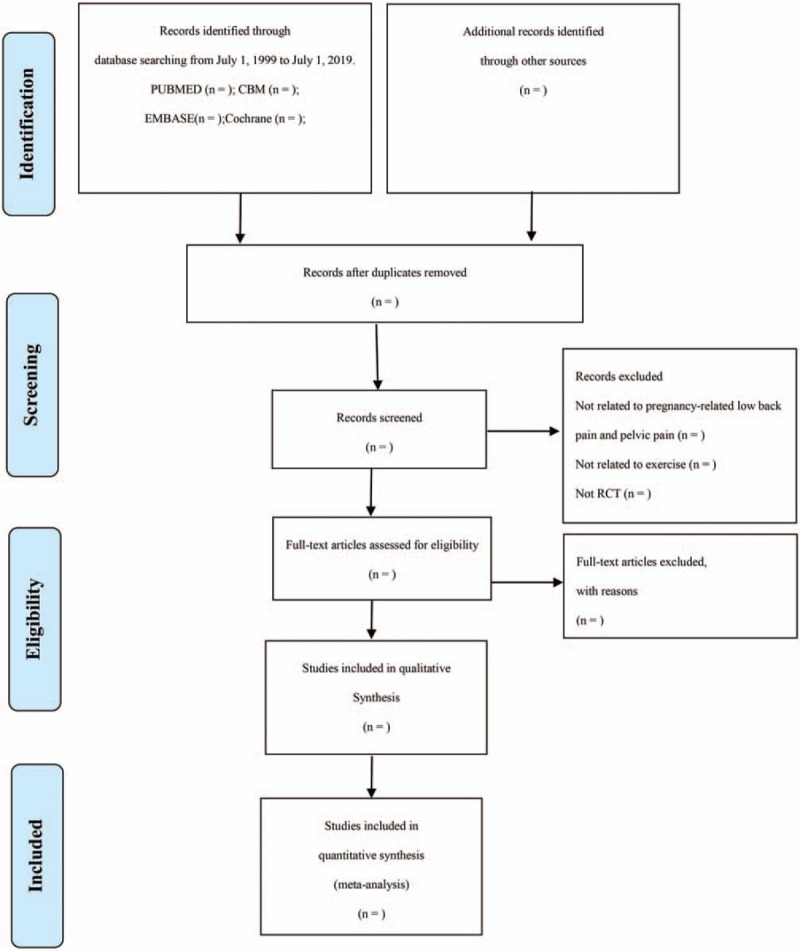
Flow diagram of study selection. CBM = Chinese BioMedicial Literature Database, RCT = randomized controlled trial.

First of all, 2 review authors (XZ and LX) will independently examine the titles and abstracts of the search results and make a preliminary selection of possible articles. The Endnote X7 software will be used to record and manage them. Secondly, through continuous reading of the full text of the preliminary selective papers, 2 independent reviewers select eligible studies on the basis of our predetermined inclusion criteria. Finally, the articles selected by 2 independent reviewers will be sorted out after the same contents are removed. If 2 articles are on behalf of duplicate publications of a study, only the 1 with the most complete data will be included. To resolve differences regarding inclusion or exclusion, 2 independent reviewers will first discuss with each other and then negotiate with another experienced reviewer (YL). All eligible studies will be included in qualitative and/or quantitative analyses. Details of the entire selection process are shown in a PRISMA flow chart^[24]^ (Fig. 1).

#### 2.2.2 Data and information extraction

We will make a detailed data and information extraction table (Table 2), which mainly includes the following items:

(1) Published materials (first author's name, contact information, year, country and region);
(2) Participants’ characteristics (source, sample size, mean age, race ratio, LBP and PP duration, lesion side, LBP and PP type and severity, use of other treatments for daily living or sleep disorders);
(3) Intervention measures (ET styles, frequency of each training, time of each training, total training time);
(4) Comparison (treatment modes and types, frequencies, time or dose per treatment, course of treatment);
(5) Outcomes and others (scale tools, evaluation time, outcome details, informed consent, adverse events, drop-out rates and causes, costs and funding sources);
(6) Study design (randomized, blinded).

**Table 2:**
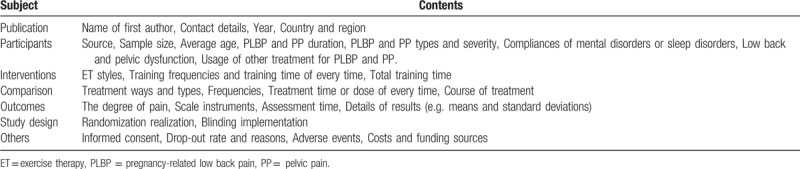
Data and information extraction schedule.

The above information or data will be obtained through reading the full text and contact the original investigator for confirmation. Data and information management will use Microsoft Excel 2013.

#### 2.2.3 Dealing with missing data

The missing data may influence research results to some extent and even lead to different research conclusions. Therefore, in the process of data extraction, we will contact the author of the article or the original researcher to determine whether there is any missing data in each included study. If there is missing data, we will further examine and record how they are processed in the statistical analysis, and evaluate whether their methods are reasonable. If the processing method is unlikely to significantly distort the statistical results, we will combine their data. Otherwise, we will have to stop synthesizing these data to reduce bias. For a small number of research results lacking standard deviation, we will try to get from the original researchers. If the attempts fail, we will attempt to fix them by borrowing the standard deviations of the most similar studies. Importantly, we will analyze and report on the potential impact of missing or incomplete data in the summary results.

#### 2.2.4 Appraisal of study quality

**Figure 2:**
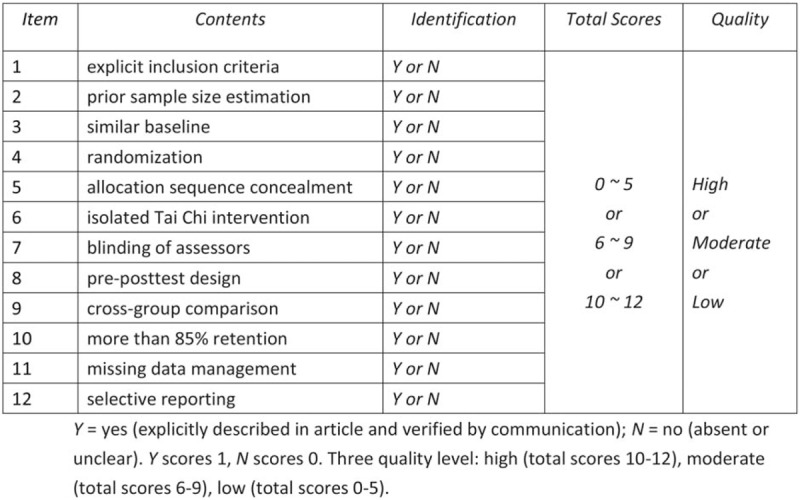
Modified assessment form. Y = yes (explicitly described in article and verified by communication); N = no (absent or unclear). *Y* scores 1, *N* scores 0. Three quality level: high (total scores 10–12), moderate (total scores 6–9), low (total scores 0–5).

In view of the specificity of ET interventions, we developed a revised assessment form based on the Cochrane tool bias risk and physiotherapy evidence database (PEDro) scale to assess methodological quality of eligible studies. The revised evaluation form mainly includes the following 11 items: item 1= clear inclusion criteria; item 2 = prior sample size estimation; item 3 = similar baseline; item 4 = randomization; item 5 = hidden order of assignment; item 6 = ET isolated intervention; item 7 = blind jurors; item 8 = pre-posttest design; item 9 = cross-domain comparisons; item 10= retention rate over 85%; item 11= management of missing data (if missing data exists); item 12 = selective reporting. Each item will be graded as Y = yes (clearly described in the article and verified by communication), or N = no (absent or unclear). The *Y* value of the project identification is 1, and the *N* value of the project identification is 0. According to the total score, each study was divided into 3 quality levels: high (10–12 points), medium (6–9 points) and low (0–5 points). The details of the qualitative assessment are shown in Figure 2.

Before the appraisal of the above methodology project, 2 independent reviewers (XZ and LX) communicate and verify with the original author in advance to avoid misjudgment. As the primary basis for evaluating the quality and classification of research, all responses or explanations of the original authors are recorded in detail. Any differences will be resolved through discussion and negotiation with a third experienced reviewer (YL).

#### 2.2.5 Assessment of reporting bias

If there are no <10 studies available for quantitative analysis, we will generate funnel plots to assess reported bias. For continuous variables, the Egger test will also be adopted to check the asymmetry of funnel plots. However, even if the test does not provide evidence of funnel plot asymmetry, reporting bias (including publication bias) cannot be excluded due to the relatively low testing capacity. Asymmetric funnel plots are generally considered to have publication bias, which is a type of reporting bias, but it also implies that there may be other causes, such as differences in methodological quality or true heterogeneity of intervention effects. We will analyze the possible reasons and give a reasonable explanation for the asymmetric funnel plot.

#### 2.2.6 Assessment of heterogeneity

Heterogeneity evaluation included 2 heterogeneity tests, *x*^2^ test (significance level: 0.1) and *I*^2^ test. The former checks for heterogeneity, while the latter reflects the degree of heterogeneity through a specific value (typically 25% or less = low, 25%–75%= medium, 75% or more = high). When high heterogeneity occurs, we will analyze its possible sources.

#### 2.2.7 Measure of treatment effect

For dichotomous variables such as adverse events, we will calculate the risk ratio or odds ratio with 95% confidence interval (CI). For continuous variables, we will calculate the mean difference from 95% CI or the standard mean difference.

#### 2.2.8 Data synthesis

Quantitative synthesis will be carried out after qualitative analysis. Qualified studies with complete and no missing data will be quantitatively synthesized. It will also include studies of incomplete data for quantitative synthesis where data can be retrieved or reasonably repaired. Only qualitative analysis can be carried out for the research that has been existed with incomplete data and/or unreasonable methods for processing missing data. Quantitative data synthesis will be carried out by Review Manager software (Revman5.3, available from the Cochrane Web site http://tech.cochrane.org/Revman). If the *I*^2^ value is no >50%, indicating relatively small heterogeneity, the fixed effect model should be used to obtain the comprehensive results. Otherwise, the random effect model will be used.

#### 2.2.9 Subgroup analysis

Considering the possibility of high heterogeneity, we will conduct a subgroup analysis project to get an objective conclusion. First, data of participants in different recovery periods (within 1 month, 2–6 months, and 6 months or more) will be analyzed. Second, data of different comparative designs, such as ET and blank control, ET and conventional rehabilitation therapy (CRT), combined application of ET and CRT, will be analyzed. Thirdly, if possible, analyze the data separately for different ET styles, training times, and frequencies. In addition, heterogeneity may be higher due to factors such as quality of test methodology, age, lesion site or nature, severity, ability to live daily or sleep disorders. These factors need to be considered in subgroup analysis.

#### 2.2.10 Sensitivity analysis

After data synthesis, we plan to conduct sensitivity analysis by excluding combined studies 1 by 1 to observe whether there is significant change in the comprehensive results. Significant changes are reflected in studies that are sufficient to affect the overall synthesis results, so it is necessary to reevaluate them and make a careful decision whether to merge or not. We must give a reasonable reason before we make a decision. If there is no significant change, we can assume that our overall results are firm.

#### 2.2.11 Quality of evidence

An internationally recognized scoring system will be used to assess the quality of our evidence. We will use GRADEpro3.6 software to qualitatively evaluate the level of evidence. Considering the fact that only RCT is accepted, we will downgrade the quality of the evidence model, which involves the following 5 factors: risk of bias, inconsistency, indirectness, inaccuracy, and publication bias. The level of evidence will be high, medium, low and very low.

## 3 Discussion

As the saying goes: “Exercise is the medicine.” Exercises such as a pelvic exercise program with pelvic realignment device, yoga, pilates, sling, bobath balls, aerobic, and resistance exercises, and so on, are often recommended for low back pain and PP.^[20]^ However, few high-quality studies could provide strong evidence about their efficacy and safety. Investigators have made some systematic reviews or meta-analyses to get comprehensive evidence in recent years. A meta-analysis demonstrated that compared to general exercise, core stability exercise is more effective in decreasing pain and may improve physical function in patients with chronic LBP in the short term.^[25,26]^ Another RCT showed that effects of exercise with a pelvic realignment device is more effective than core stability exercise in the short term for PLBP and PP.^[27]^ Effects of noninvasive management on function and disability were mixed. Future studies should identify which sub-groups of PLBP and PP respond to specific interventions.^[28]^ A meta-analysis of manual therapy and exercise showed that combining different forms of manual therapy with exercise is better than manual therapy, nevertheless, future RCTs should be more rigorous in their investigation by not mixing categories of patients as well as intervention types.^[29]^ Therefore, this paper conducted a meta-analysis on the treatment of PLPB and PP with exercises, providing more reliable evidence for future studies. It is noteworthy that this study has subgroup analysis according to the types of different exercises, which may lead to relative specific conclusions. To the best of our knowledge, there has been no one meta-analysis specially analyzing ET's effect for PLBP and PP. We hope to provide more practical and targeted results investigating the effect of ET for PLBP and PP in the current systematic review and meta-analysis.

As is known, the key to achieve a reliable meta-analysis result lies in incorporating sufficient data from high-quality original literature and perform rigorous methodological quality assessment. Allowing for the particularity of ET, we make a modified assessment form which incorporates the advantages of Cochrane assessment tool and PEDro scale, making our qualitative evaluation more reasonable and practical. And also, it is sensible that our quality assessment will not only include reading original articles to know methodological execution but also making verification with original authors to reduce the possibility of misjudgment.

The strengths of our study mainly include that comprehensive searching for Chinese and English databases, rigorous evaluation of quality, and sensible subgroup analysis design, all of which will make our analysis result more convictive. One limitation of this review is that we will only search Chinese and English databases, possibly missing some articles published using other language. Another limitation is that the large heterogeneity may emerge, leading to adverse effect on the final conclusion.

## Author contributions

Xiang Hu, Xianghu Zhao, Zengbin Zheng, and Liang Xu conceived the study.

The protocol was drafted by Xianghu Zhao, Zengbin Zheng, and revised by Ming Ma and Wudong Sun. Xianghu Zhao and Ming Ma developed the search strategy. Xianghu Zhao and Liang Xu will independently work on study selection, quality assessment, data extraction, and synthesis.

**Conceptualization:** Xianghu Zhao, Xiang Hu, Wudong Sun, Yanli Liu.

**Data curation:** Liang Xu, Zengbin Zheng.

**Formal analysis:** Xianghu Zhao.

**Funding acquisition:** Ming Ma, Wudong Sun.

**Investigation:** Zengbin Zheng, Liang Xu.

**Methodology:** Xianghu Zhao, Ming Ma, Yanli Liu.

**Software:** Liang Xu, Zengbin Zheng.

**Supervision:** Ming Ma.

**Writing – original draft:** Xiang Hu, Xianghu Zhao.

**Writing – review and editing:** Ming Ma, Xiang Hu.
